# Pseudoaveraging for denoising of OCT angiography: a deep learning approach for image quality enhancement in healthy and diabetic eyes

**DOI:** 10.1186/s40942-023-00486-5

**Published:** 2023-10-11

**Authors:** Omar Abu-Qamar, Warren Lewis, Luisa S. M. Mendonca, Luis De Sisternes, Adam Chin, A. Yasin Alibhai, Isaac Gendelman, Elias Reichel, Stephanie Magazzeni, Sophie Kubach, Mary Durbin, Andre J. Witkin, Caroline R. Baumal, Jay S. Duker, Nadia K. Waheed

**Affiliations:** 1 New England Eye Center, Tufts Medical Center, 800 Washington St., Box 450, Boston, MA 02111 USA; 2 Research and Development, Carl Zeiss Meditec, Dublin, CA 94568 USA; 3 Department of Ophthalmology, Federal University of Sao Paulo, Sao Paulo, Brazil; 4 Boston Image Reading Center, 55 Causeway street, Boston, MA 02114 USA

**Keywords:** Averaging, Image artifact, Deep learning, Denoising, Diabetic retinopathy, Image quality, Optical coherence tomography angiography, OCTA, Pseudoaveraging

## Abstract

**Background:**

This study aimed to develop a deep learning (DL) algorithm that enhances the quality of a single-frame enface OCTA scan to make it comparable to 4-frame averaged scan without the need for the repeated acquisitions required for averaging.

**Methods:**

Each of the healthy eyes and eyes from diabetic subjects that were prospectively enrolled in this cross-sectional study underwent four repeated 6 × 6 mm macular scans (PLEX Elite 9000 SS-OCT), and the repeated scans of each eye were co-registered to produce 4-frame averages. This prospective dataset of original (single-frame) enface scans and their corresponding averaged scans was divided into a training dataset and a validation dataset. In the training dataset, a DL algorithm (named pseudoaveraging) was trained using original scans as input and 4-frame averages as target. In the validation dataset, the pseudoaveraging algorithm was applied to single-frame scans to produce pseudoaveraged scans, and the single-frame and its corresponding averaged and pseudoaveraged scans were all qualitatively compared. In a separate retrospectively collected dataset of single-frame scans from eyes of diabetic subjects, the DL algorithm was applied, and the produced pseudoaveraged scan was qualitatively compared against its corresponding original.

**Results:**

This study included 39 eyes that comprised the prospective dataset (split into 5 eyes for training and 34 eyes for validating the DL algorithm), and 105 eyes that comprised the retrospective test dataset. Of the total 144 study eyes, 58% had any level of diabetic retinopathy (with and without diabetic macular edema), and the rest were from healthy eyes or eyes of diabetic subjects but without diabetic retinopathy and without macular edema. Grading results in the validation dataset showed that the pseudoaveraged enface scan ranked best in overall scan quality, background noise reduction, and visibility of microaneurysms (*p* < 0.05). Averaged scan ranked best for motion artifact reduction (*p* < 0.05). Grading results in the test dataset showed that pseudoaveraging resulted in enhanced small vessels, reduction of background noise, and motion artifact in 100%, 82%, and 98% of scans, respectively. Rates of false-positive/-negative perfusion were zero.

**Conclusion:**

Pseudoaveraging is a feasible DL approach to more efficiently improve enface OCTA scan quality without introducing notable image artifacts.

**Supplementary Information:**

The online version contains supplementary material available at 10.1186/s40942-023-00486-5.

## Background

Optical coherence tomography angiography (OCTA) is an imaging modality that generates images of the retinal and choroidal vasculature by repeatedly scanning one area and inferring the presence of blood flow between sequential images based on the decorrelation signal [1]. Sequential scanning of the same area increases the acquisition time in comparison to single acquisition and leads to a higher incidence of artifacts such as motion artifact [2]. The presence of such artifacts and noise may affect image interpretation and impair quantitative image analyses [3]. In this context, strategies to improve imaging quality are needed to overcome such artifacts.

Several hardware and software strategies have been employed to overcome imaging artifacts on OCTA and to optimize the signal-to-noise ratio, such as the implementation of active eye tracking and averaging of images [1]. By registering repeated OCTA scan acquisitions, averaging achieves higher signal-to-noise ratio and improves the visualization of the microvasculature in healthy eyes, as well as eyes with retinal pathology [4–10]. Although proven to be effective, averaging is a time consuming strategy that requires post-acquisition processing and registration of the repeated images [11]. Kadomoto et al. showed that the time spent to obtain a 10-frame averaged image was nearly 17 times the time required for a single acquisition [11, 12]. Of note, averaging in Kodomoto’s study was done as a built-in feature, so even more time is expected for systems where averaging is done as a separate process.

Deep learning (DL) is another potential strategy where an algorithm can be trained to construct high-quality scans from lower-quality scans [13, 14]. Such denoising DL algorithms were extensively studied in the field of neuroimaging [15]. In one study an algorithm was trained to enhance low-dose gadolinium contrast magnetic resonance images of the brain to make it appear comparable to full-dose scans, thereby achieving diagnostic quality images with gadolinium doses tenfold lower than those typically used [16]. Similarly, a spectral domain (SD) OCTA DL denoising algorithm has been reported, comparing single OCTA enface 3 × 3 mm scans to 4-frame averaged enface scans, showing improvements in signal-to-noise ratio and pathology detection. [11, 12, 17]

Swept-source (SS) optical coherence tomography (OCT) instruments, with their higher scan speeds, allow for faster acquisition times and larger scanning areas compared to SD-OCT devices [18]. As SS-OCT instruments continue to become more widely available, strategies to improve image quality on SS systems are also necessary. To the best of our knowledge, DL denoising strategies have not yet been applied to OCTA scans obtained by a SS instrument. Therefore, in this study a DL pseudoaveraging (denoising) algorithm of SS-OCTA enface scans from healthy and diabetic eyes was developed and validated by comparing the original (single-frame) scan to the 4-frame averaged and pseudoaveraged scans (the scans produced by applying the pseudoaveraging denoising algorithm to the single-frame scans). Furthermore, the algorithm’s performance was assessed for image quality enhancement and potential introduction of artifacts in a separate test dataset by comparing the original scan against the pseudoaveraged scan using a comprehensive qualitative grading of both the superficial as well as the full-thickness retina slabs.

## Methods

This cross-sectional study consists of participants presenting to the New England Eye Center (NEEC), Tufts Medical Center (TMC), Boston, Massachusetts from July 2019 through March 2020. Informed consent was obtained from all participants. This study received approval by the institutional review board and abided by the Declaration of Helsinki and the Health Insurance Portability and Accountability Act.

### Study population

This study is composed of a prospective dataset (split into a training dataset and validation dataset) and a retrospective test dataset.

### Prospective dataset

Eyes included in the prospective dataset were of self-reported healthy participants or those with diabetes and any level of diabetic retinopathy (DR) with or without diabetic macular edema (DME). Patients underwent a comprehensive ophthalmic examination including imaging, in which the absence of any retinal diseases was confirmed for self-reported healthy participants, and the presence of DR and DME, and level of DR were assessed for diabetic participants. All study eyes in the prospective dataset underwent four repeated sequential OCTA scans using the PLEX® Elite 9000 SS-OCT (Carl Zeiss Meditec, Dublin, CA).

### Retrospective test dataset

The retrospective test dataset is composed of eyes from diabetic subjects (with and without DR and with and without DME) that had been previously imaged with a 6 × 6 mm scan centered on the fovea on the PLEX® Elite 9000 SS-OCT at the NEEC/TMC, between Jan 2017 and Jul 2018.

### Inclusion and exclusion criteria

All participants were over 18 years of age, and either or both eyes were included. Exclusion criteria included media opacity precluding acceptable quality OCTA images, concomitant non-DR macular pathology, and inability to provide a written informed consent. Unless the artifact was severe enough to render an image unreadable, eyes with artifacts were included in this study. This was to maximize generalizability and to evaluate the performance of the DL algorithm on images comparable to those obtained in a typical clinical setting. For the same reason, low scan signal strength was not an exclusion criterion.

### The SS-OCT system

The PLEX® Elite 9000 SS-OCT (Carl Zeiss Meditec, Dublin, CA) system performs at a scanning speed of 100,000 A-scans/s and has a wavelength near 1060 nm, achieving tissue axial resolution of 6.3 μm and transverse resolution of 20 μm. All OCTA scans were 6 × 6 mm centered on the fovea, and each volume had a sampling density of 500 A-scan per B-scan and a total of 500 B-scans. Segmentation in all scans was assessed and manual correction was done when needed.

### Study workflow (Fig. 1)

**Fig. 1:**
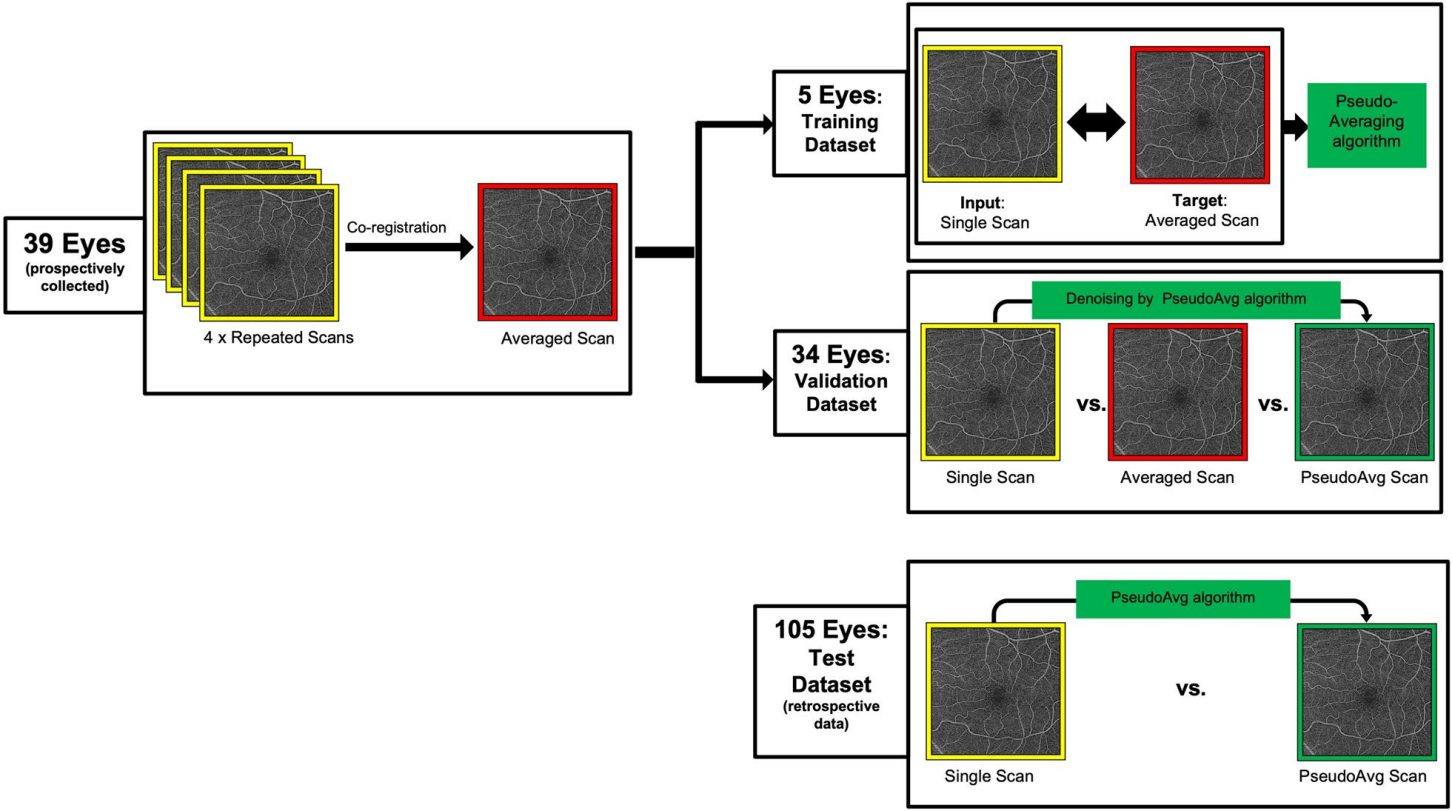
Schematic diagram illustrating the workflow of this study. The 39 prospectively collected eyes underwent 4 repeated 6 × 6 mm macular scans on the Zeiss PLEX Elite SS-OCT, and the repetitions were co-registered to generate an averaged volume. This dataset was split into a training dataset (5 eyes) and validation dataset (34 eyes). In the training dataset, the single-frame enface OCTA scan was used as an input and it was paired with its corresponding enface OCTA 4-frame average as a target to train the denoising or pseudoaveraging algorithm. Additional training of the algorithm (not shown here) was done by using 8 low-quality single-frame enface OCTA scans as an input and high-quality single-frame OCTA scan of the same eye as an output (peer-to-peer training). For the validation dataset, the pseudoaveraging algorithm was applied to the best-quality single-frame enface OCTA scan (out of the 4 repeated single-frame acquisitions), and the resulting pseudoaveraged scans were compared against their corresponding original single-frame scans as well as against their 4-frame averaged scans. In a separate test dataset of 105 eyes retrospectively collected, the algorithm was applied to single-frame enface OCTA scans, and the original and its corresponding pseudoaveraged enface scans were compared

We split the prospectively acquired data into a training dataset, selected based on good-quality corresponding averages, and a validation dataset to train and validate the performance of the denoising or pseudoaveraging algorithm, respectively. The retrospective dataset served as a separate test dataset. The workflow of this study is depicted in Fig. 1 and described in detail in Fig. 1.

### Averaging and pseudoaveraging (Denoising)

#### Averaging

The 4-frame averages were created using a custom-made program written in C++/C# language that uses both structural and flow data by registering each of the four repetitions to a reference, topographically—using common key points observed in flow data projections, and axially—using retinal layer information in the structural data. For simplicity, the enface OCTA projection/scan from the 4-frame averaged volume will be referred to as the “averaged” scan and will be used interchangeably with “4-frame averaged” scan throughout this paper.

### Pseudoaveraging (Denoising)

To create the pseudoaveraged scans, a 5-layer U-net convolutional neural network with stochastic gradient descent optimization was used. The pseudoaveraging algorithm was trained using the single-frame enface OCTA scan as input and its’ corresponding 4-frame averaged enface OCTA scan as a target. The single-frame input scan was selected as the one with the best quality out of the 4 repeated single-frame acquisitions for each eye in the training dataset. Since the averaged scan was used as the “target” scan to train the algorithm, we selected the scans for the training dataset as the ones with best averaged scans' quality. The averaged scan quality was evaluated based on subjective assessment of the visibility and quality of clinically relevant image features such as appearance of small vessels and presence of background noise. Moreover, although the scans selected for training were among the best in terms of overall averaged scan quality, in some instances a small region or “a patch” of the averaged scan was affected by artifact (e.g., a small region of the averaged scan had low signal strength due to shadowing from media opacity, or was impacted by motion artifact). In order to prevent training the DL model from viewing these patches with artifact as “targets” for training, these regions in the target averaged scans were manually masked. Lastly, besides the “single-to-average” pairs training, additional “peer-to-peer” training was done by using low-quality single-frame enface OCTA scan as an input and high-quality single-frame OCTA scan of the same eye as an output. This was done to improve the ability of the algorithm to process images with regions of low quality or low signal without rendering them as apparently ischemic. In other words, to prevent the algorithm from erroneously identify these regions as noise or artifact and subsequently denoising them. After training, the DL algorithm was applied to single-frame enface OCTA scans in the validation and test datasets, and the outputted pseudoaveraged scans were compared against other scans as detailed later under grading section.

### Architecture of the pseudoaveraging algorithm

The pseudoaveraging algorithm is a convolutional neural network that is a 5-layer U-net operating on 64 × 64 patches from a single-frame scan, trained against corresponding patches of 4-frame averaged scan of the same eye (single-to-averaged pairs), so each scan consisted of approximately 1000 patches, and this can be considered the effective training dataset sample size, which is a number significantly larger than the seemingly small training dataset sample size if one to only consider the number of scans as the training dataset sample size. This strategy relies on the use of data augmentation, allowing for network end-to-end training from very few images [19]. The implementation of the training strategy in our study was similar to that in Ronnenberger et al., but using an L1 norm of the difference with the ground truth as a cost function instead of soft-max/cross entropy. [19]

### OCTA enface slab selection

In our training, validation, and testing procedure, we used the OCTA *enface* projections, and we used the PLEX® Elite preset slabs defined as follows: the preset superficial slab is defined by the internal limiting membrane as its upper boundary, and the inner plexiform layer as its lower boundary. The preset full-thickness retina slab is defined by the inner limiting membrane as its inner slab boundary and an offset above the RPE-fit by 70 μm as its outer boundary.

The PLEX® Elite superficial slab was used for training the pseudoaveraging algorithm. The reason for choosing the superficial slab for training the algorithm is that the averaging function we used performed better in the superficial slabs compared to the full-thickness retina slabs, because averaging of the denser retina slabs resulted in tiny errors in registration that caused a decrease in contrast in the finest vessels of the averaged retina scan (unpublished data). We validated the model performance using the full-thickness retina slabs in the validation dataset and tested the model’s performance using both the superficial and full-thickness retina slab in the test dataset. Although the model was trained and validated using different slabs, the slabs are not really distinct since the superficial slab is essentially part of the full-thickness retina slab as defined above.

### Scans grading (ranking)

For validating and testing the model’s performance, our ultimate metric that we chose was the grader evaluation of image quality, rather than a numerical measure of image quality; the latter is often more objective and easier to calculate, but can correlate less well with actual utility. The grading was done independently by two graders masked to scans labels. The two graders have expertise in imaging interpretation and are active in clinical ophthalmology.

### Grading the validation dataset

For grading eyes in the validation dataset, a single, an averaged, and a pseudoaveraged scan of each eye were ranked by two masked graders (OAQ, LSMM). All images in the validation dataset were full-thickness retinal enface OCTA slabs. Grading was done using a Mac operating system with the default Preview software. The two masked graders independently ranked images based on a subjective qualitative grading system that evaluated scan’s quality metrics (overall scan enhancement, background noise reduction, motion (line) artifact reduction, appearance of small vessels, and continuity of the foveal avascular zone [FAZ] contour) and pathologic features visualization metrics (appearance of areas of non-perfusion and pruned vessels, and visibility of microaneurysms [MA]). A detailed definition of all grading metrics is provided in Additional file 2: Table S1. Ranking score was 1 through 3, with a score of 3 being the best rank, relative to the other two masked scans for each of the grading metric defined above. Equal ranking was allowed when two or more images appeared similar and the difference was not clinically meaningful (i.e., not expected to impact image interpretability or features visualization). Open adjudication was performed whenever scans were ranked differently between the two masked graders until agreement was reached in all cases. Senior study member (NKW) was available in case agreement was not reached.

### Grading the test dataset

For grading eyes in the test dataset, two enface OCTA scans (single and pseudoaveraged) were compared, and additional metrics evaluating the presence of false-positive/-negative perfusion and the presence of artifacts introduced by pseudoaveraging were included (Additional file 2: Table S1). The superficial slab and the full-thickness retina slab were used in the test dataset grading.

### Statistical analysis

Demographics were summarized using summary statistics. For the validation dataset, the Wilcoxon Signed Rank test was selected as the test of choice to compare the ranking of images in the validation dataset since this test is a non-parametric test that is appropriate for paired data (pre- and post-processed scans of the same eye). Results from grading the test dataset were reported using summary statistics (absolute numbers and percentages of scans that showed improvement or presence of an artifact when comparing the pseudoaveraged to the original scan). A two-sided *p*-value of < 0.05 was considered statistically significant for these exploratory analyses.

## Results

Eighty four participants were included with a mean (SD) age of 55 (15.9) years old, and 45% were female (Table 1). Of their 144 included eyes, approximately 42% were healthy eyes or from diabetic subjects but without DR, 24% had mild non-proliferative DR (NPDR), 10% had moderate NPDR, 7% had severe NPDR, and 17% had proliferative DR (Table 1). Five eyes were included in the training dataset, 34 eyes were used as a validation dataset, and 105 eyes were used as a separate test dataset (Table 1, Fig. 1).

**Table 1:**
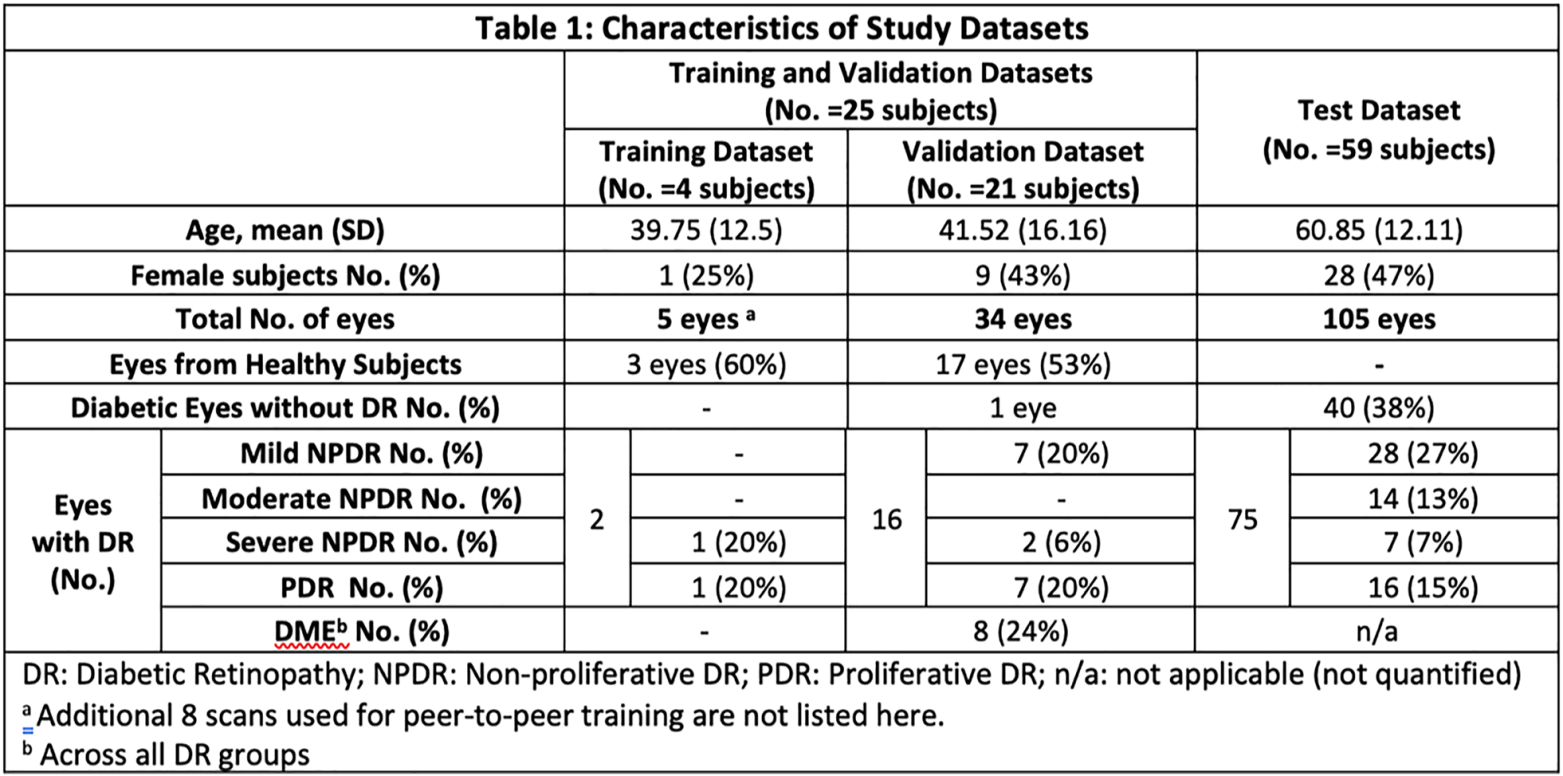
Characteristics of study datasets

Blue stained embryos were recorded as GUS +  and unstained embryos as GUS−

### Validation dataset

**Fig. 2:**
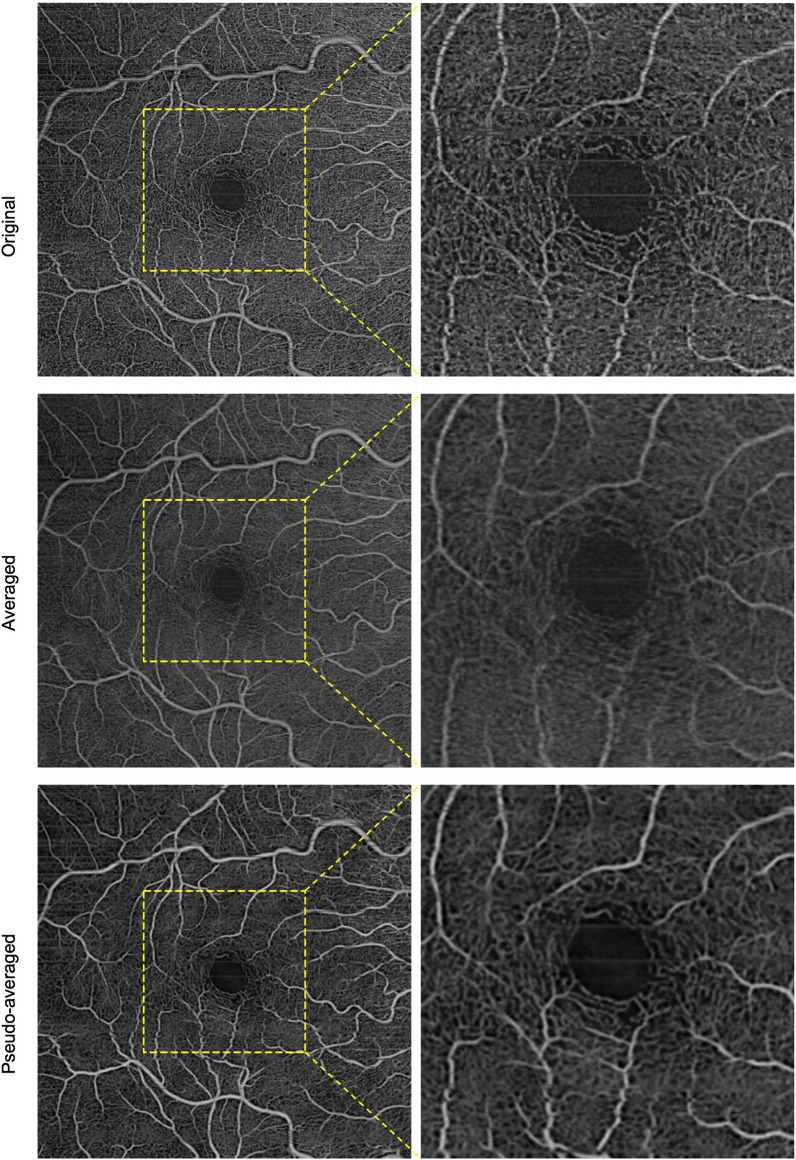
Example of a single (top), an averaged (middle), and a pseudoaveraged (bottom) full-thickness retinal enface OCTA scan in a healthy eye. It is noted how the overall scan quality, small vessels continuity, and background noise reduction are best in the pseudoaveraged scan. It is noted how the averaged scan is the best for motion (line) artifact correction;s however, the small vessels appear blurry in this scan (insets)

In the validation dataset, the full-thickness retina slabs of the original, averaged, and pseudoaveraged (denoised) scans were ranked and grading showed that for overall scan quality, background noise reduction and appearance of areas of non-perfusion/pruned vessels and for visibility of microaneurysms, the pseudoaveraged scan ranked best (*p* < 0.05). The averaged scan ranked best for motion (line) artifact reduction and lowest for appearance of small vessels and FAZ contour continuity (*p* < 0.05) (Table 2) (Fig. 2 and Additional file 1: Fig. S1).

**Table 2:** Validation dataset grading results

	Grading Metrics	PseudoAvg vs. original (better ranked scan)		PseudoAvg vs. averaged (better ranked scan)		Averaged vs. Original (better ranked scan)	
Scan Quality Metrics	Overall Scan Enhancement	PseudoAvg	*p* < 0.0001	PseudoAvg	*p* < 0.0001	Equal	*p* = 0.4575
	Background Noise Reduction	PseudoAvg	*p* < 0.0001	PseudoAvg	*p* < 0.0001	Equal	*p* = 1.0
	Motion (line) Artifact Reduction	PseudoAvg	*p* = 0.031	Averaged	*p* < 0.0001	Averaged	*p* < 0.0001
	Small Vessels Appearance	PseudoAvg	*p* < 0.0001	PseudoAvg	*p* < 0.0001	Original	*p* < 0.0001
	Continuity of FAZ Contour	Equal	*p* = 0.625	PseudoAvg	*p* = 0.0013	Original	*p* = 0.0011
Pathologic features Metrics	Appearance of Non-perfused Areas	PseudoAvg	*p* = 0.032	PseudoAvg	*p* = 0.0098	Equal	*p* = 0.5058
	Appearance of Pruned Vessels	PseudoAvg	*p* = 0.001	PseudoAvg	*p* = 0.0078	Equal	*p* = 0.1250
	Visibility of MAs	PseudoAvg	*p* = 0.0005	PseudoAvg	*p* = 0.0002	Equal	*p* = 0.0625

Comparisons and *p*-values using the Wilcoxon Signed Rank Test; PseudoAvg: Pseudoaveraged scan; FAZ: Foveal Avascular Zone; MA: microaneurysms

### Test dataset

**Fig. 3:**
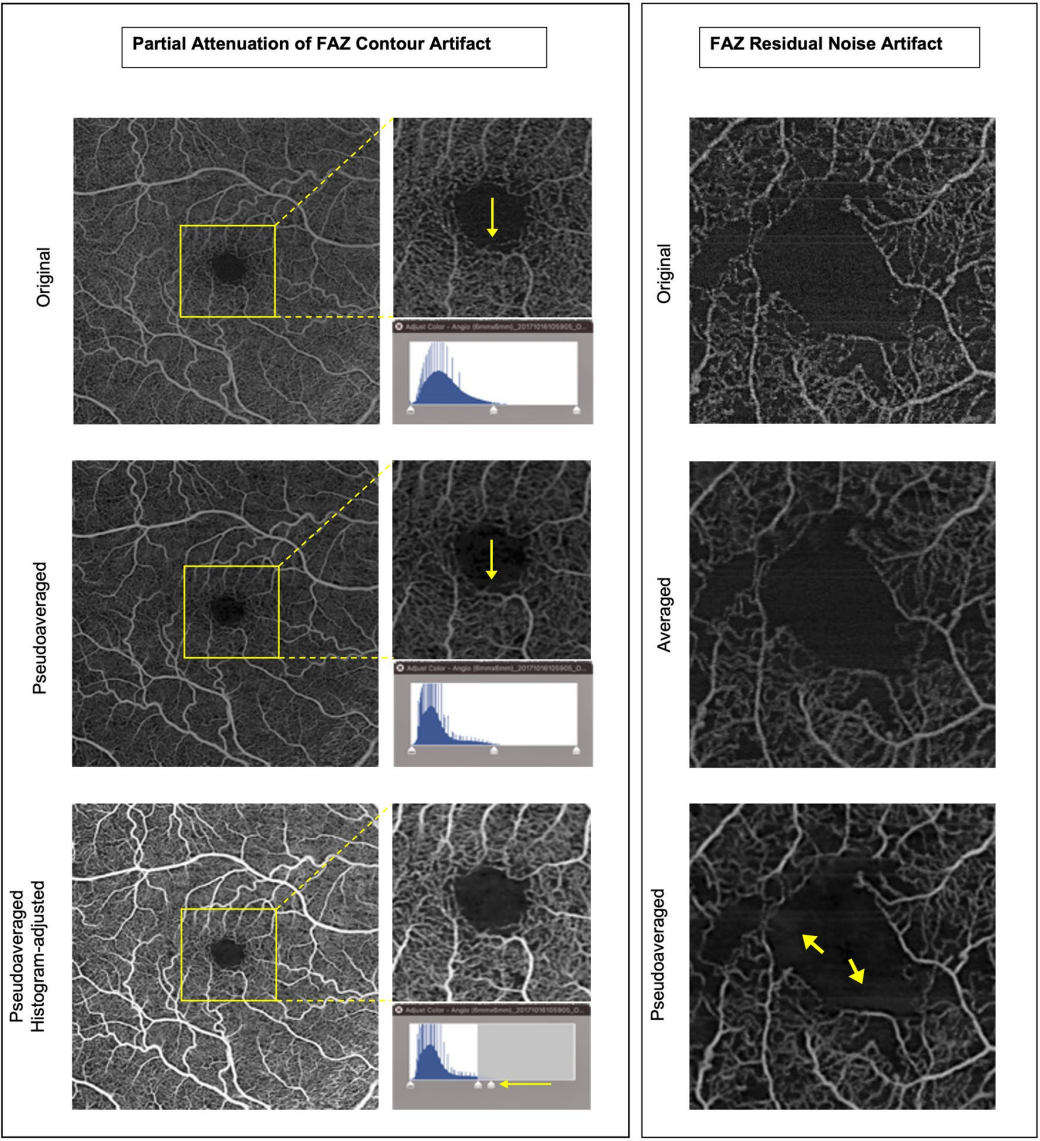
Left panel: 6 × 6 mm enface OCTA scan demonstrating partial attenuation of FAZ contour in the pseudoaveraged scan at about 6 o’clock (middle) compared to the original scan (top). However, after adjusting the brightness/contrast histogram in image J, the vessel is no longer as attenuated and FAZ contour becomes more visible (bottom image) indicating that FAZ contour attenuation is at least partially due to inadequate brightness/contrast adjustment and not only due to pseudoaveraging. Right panel: cropped 6 × 6 mm enface OCTA scan from an eye with diabetic retinopathy. The arrows in the bottom image point to subtle residual FAZ noise in the pseudoaveraged scan (bottom scan)

In the test dataset, original scans were compared against their corresponding pseudoaveraged scans. Grading showed that pseudoaveraging resulted in enhancement of appearance of small vessels, reduction of background noise, and reduction (but not complete resolution) of motion artifacts in 100%, > 82%, and 98% of scans, respectively, and results were comparable in the full-thickness retina slabs and superficial slabs (Table 3). The rates of false-positive or false-negative perfusion were zero in either slab. However, a partial attenuation of FAZ contour was noted in 4.8% (5/105) of full-thickness retina slabs and 0.95% (1/105) of superficial slabs (Fig. 3). Additionally, background noise reduction of FAZ was incomplete in a subset of scans; in the full-thickness retina slabs there was residual noise that was noticeable in 27.6% and subtle in 16.2% scans, and in the superficial slab FAZ residual noise was not noticeable and was subtle in 7.6% of scans (Table 3, Fig. 3).

**Table 3:** Test dataset grading results

	Grading Metrics	Proportion of scans after pseudoaveraging, No. (%)	
		Full-thickness retina slab (No. = 105)	Superficial slab (No. = 105)
Scan Quality Metrics	Enhanced Small Vessels Appearance, No. (%)	105 (100%)	105 (100%)
	Motion (line) Artifact Reduction, No. (%)	56/68 scans (82%)	64/75 scans (85%)
	Background Noise Reduction, No. (%)	103 (98%)	103 (98%)
Artifact Assessment Metrics	False-Negative Perfusion, No. (%)	0 (0%)	0 (0%)
	False-positive perfusion, No. (%)	0 (0%)	0 (0%)
	Partial Attenuation of FAZ Contour, No. (%)	5 (4.8%)	1 (0.95%)
	FAZ Residual Noise Artifact		
	Noticeable, No. (%)	29 (28%)	0 (0%)
	Subtle, No. (%)	17 (16%)	8 (7.6%)

FAZ: Foveal avascular zone

## Discussion

In this cross-sectional study, we developed and validated a DL algorithm that improves the quality of OCTA enface scans obtained on a SS-OCT system, and we tested the algorithm’s performance using a diverse set of scans comparable to those acquired in clinical settings. Our findings demonstrate that pseudoaveraging of single OCTA scans using a DL algorithm is a feasible and a potentially faster approach than OCTA image averaging to improve scan quality and feature visualization without the need for the repeated acquisitions required for averaging and without introducing notable image artifacts that may appear after denoising.

Pseudoaveraged scans were superior to single OCTA scans as well as, surprisingly, averaged OCTA scans in most grading parameters. One reason why averaged OCTA scans were of lesser quality than pseudoaveraged scans may be due to imperfect co-registration of the repeated acquisitions that make up an averaged scan. Additionally, the quality of an averaged scan is dependent on the quality of each of its constituent acquisitions, and since acquiring repeated scans increases the imaging time and procedural complexity, the quality of the repetitions may be suboptimal—especially when imaging older subjects with ocular opacities, poor fixation, and ocular fatigue. We believe that for these reasons the averaged scan ranked lowest for continuity of small vessels and FAZ contour appearance.

The only exception where the averaged scan was superior to the other scans was in motion (line) artifact correction. Compared to the original scan, the pseudoaveraged scan reduced line artifacts but did not completely resolve them. Averaging, on the other hand, uses data from multiple acquisitions and is able to compensate for regions affected by motion utilizing unaffected repeated scans, therefore allowing for more complete removal of line artifacts.

Results from the test dataset demonstrated significant improvement in the quality of superficial and full-thickness retina slabs after pseudoaveraging without introducing notable image artifacts such as false-positive or false-negative perfusion, which may theoretically occur as a result of denoising by DL algorithms. Of note, earlier iterations of the presented pseudoaveraging algorithm tended to interpret regions in the original scan with low signal as noise and subsequently denoised these regions, causing them to appear as false-negative perfusion (not shown here). In contrast, the denoising algorithm reported by Kadomoto and Kawai et al. seemed to also err on the side of false-positive perfusion (in up to 5.7% of scans), where noise was turned into “fictional vessels” [11, 12]. We were able to bring the rate of false-negative perfusion artifact to zero by training the presented algorithm with careful selection of high-quality original-averaged pairs for the training dataset, manually masking regions of low signal in the scans in the training dataset, peer-to-peer training (pairs of poor-to-good quality single-frame images), and matching the brightness/contrast pre- and post-denoising. The tendency of the earlier iteration of our presented pseudoaveraging algorithm to over-denoise compared to the ones published by other groups highlights that care should be taken when developing a DL denoising or pseudoaveraging algorithm to correctly identify noise as such and remove it without over- or under-denoising. The approaches we used in our training can be used as a strategies to overcome such artifacts when it arise.

The presented denoising algorithm had limitations. Firstly, there was partial attenuation of FAZ contour in a few retina projection scans (5/105) and in one superficial projection scan (1/105). Secondly, the background noise reduction in FAZ was sometimes incomplete and residual noise was noticeable in a subset of full-thickness retina slabs but appeared very subtle in a few superficial slabs. Nonetheless, the FAZ contour attenuation was limited to about one clock hour or less and the full contour of FAZ could still be delineated in all cases and, as shown in Fig. 3, the attenuation in some cases may be at least partially due to inadequate brightness/contrast adjustment rather than due to pseudoaveraging. Likewise, the residual FAZ noise was subtle in most cases and essentially cosmetic. Both flaws do not affect image interpretability, which makes them relatively minor. The slightly higher rates of these flaws in the full-thickness retina slabs suggest that the presented algorithm performs better in the superficial slab compared to the full-thickness retina slab. This is maybe partially explained by the fact that the algorithm was trained using the superficial slab. Additionally, the full-thickness retina slab is thicker with denser capillary beds and more noise which render it more challenging to denoise.

This study’s strengths include its relatively large and diverse sample, including the test dataset that is composed of scans with varying quality and pathology levels making it comparable to images acquired in clinical settings. Our study used a SS-OCT system—a technology that presents advantages in comparison to a spectral domain system such as higher signal penetration, higher scanning speeds, and potentially lower motion artifact.^18^ These advantages may be further magnified when evaluating deeper layers such as the choriocapillaris. Furthermore, we used a wider OCTA scan compared to previously published work (6 × 6 vs. 3 × 3 mm), and we evaluated both the superficial and full-thickness retina slabs. Limitations included enrollment of healthy and diabetic patients only, which may limit the generalizability of this study in other retinal pathologies. Our ultimate metric that we chose to evaluate and test the model was the subjective grader evaluation of image quality, rather than a numerical measure of image quality; the latter is often more objective and easier to calculate, but can correlate less well with actual utility. Furthermore, the grading parameters are well defined in this study and are comprehensive, assessing scans’ quality and pathologic features. Moreover, grading was done by two independent masked graders (a retina specialist and a retinal imaging research fellow) using standardized monitors, and grading was followed by open adjudication.

## Conclusions

In this study using a SS-OCT system in healthy and diabetic eyes, a DL pseudoaveraging algorithm was trained and validated in a prospectively collected dataset and showed that pseudoaveraging improves scan quality and pathologic features visualization when compared to single-frame original and 4-frame averages. We tested the algorithm in a retrospective dataset and found zero rates of false-positive or false-negative perfusion and no other major image artifacts that would negatively impact clinical interpretation of the OCTA scans. Pseudoaveraging represents a promising approach to improving OCTA scans quality and utility without the need to acquire repeated scans for averaging. Future work will focus on expanding the diversity of retinal pathologies included, and it will address the impact of pseudoaveraging on quantitative OCTA metrics.

### Supplementary Information

**Additional file 1: Figure S1.** Example of a single (top), averaged (middle), and pseudoaveraged (bottom) scans in a diabetic eye. Insets (a) demonstrates improvement in scan quality, small vessels continuity, and background noise reduction, and insets (b) demonstrate improved visualization of areas of non-perfusion and pruned vessels in the pseudoaveraged scan. Insets (c) demonstrate how small vessels appear granular in the single scan (top), blurry in the averaged scan (middle), and smooth and continuous in the pseudoaveraged scan (bottom). They also demonstrate how the averaged scan (middle) is best for motion or line artifact correction (best evidenced by the continuity and smoothness of larger vessels).

**Additional file 1: Table S1.** Grading parameter's definition.

## Data Availability

Imaging data analyzed during the current study cannot be shared publicly because of protected/personal health information. However, imaging data are available from the Tufts Ethics Committee (contact via IRBOffice@tuftsmedicalcenter.org) for researchers who meet the criteria for access to confidential data. Grading results are available from the corresponding author on reasonable request.

## Abbreviations

DL: Deep learning
DME: Diabetic macular edema
DR: Diabetic retinopathy
FAZ: Foveal avascular zone
MA: Microaneurysms
NPDR: Non-proliferative diabetic retinopathy
OCT: Optical coherence tomography
OCTA: Optical coherence tomography angiography
SD: Spectral domain
SS: Swept source
