# Supplementary figures and images for: Pseudoaveraging for denoising of OCT angiography: a deep learning approach for image quality enhancement in healthy and diabetic eyes
Source: Int J Retina Vitreous. 2023 Oct 11;9:62. doi: 10.1186/s40942-023-00486-5 (PMC10568842; doi:10.1186/s40942-023-00486-5)

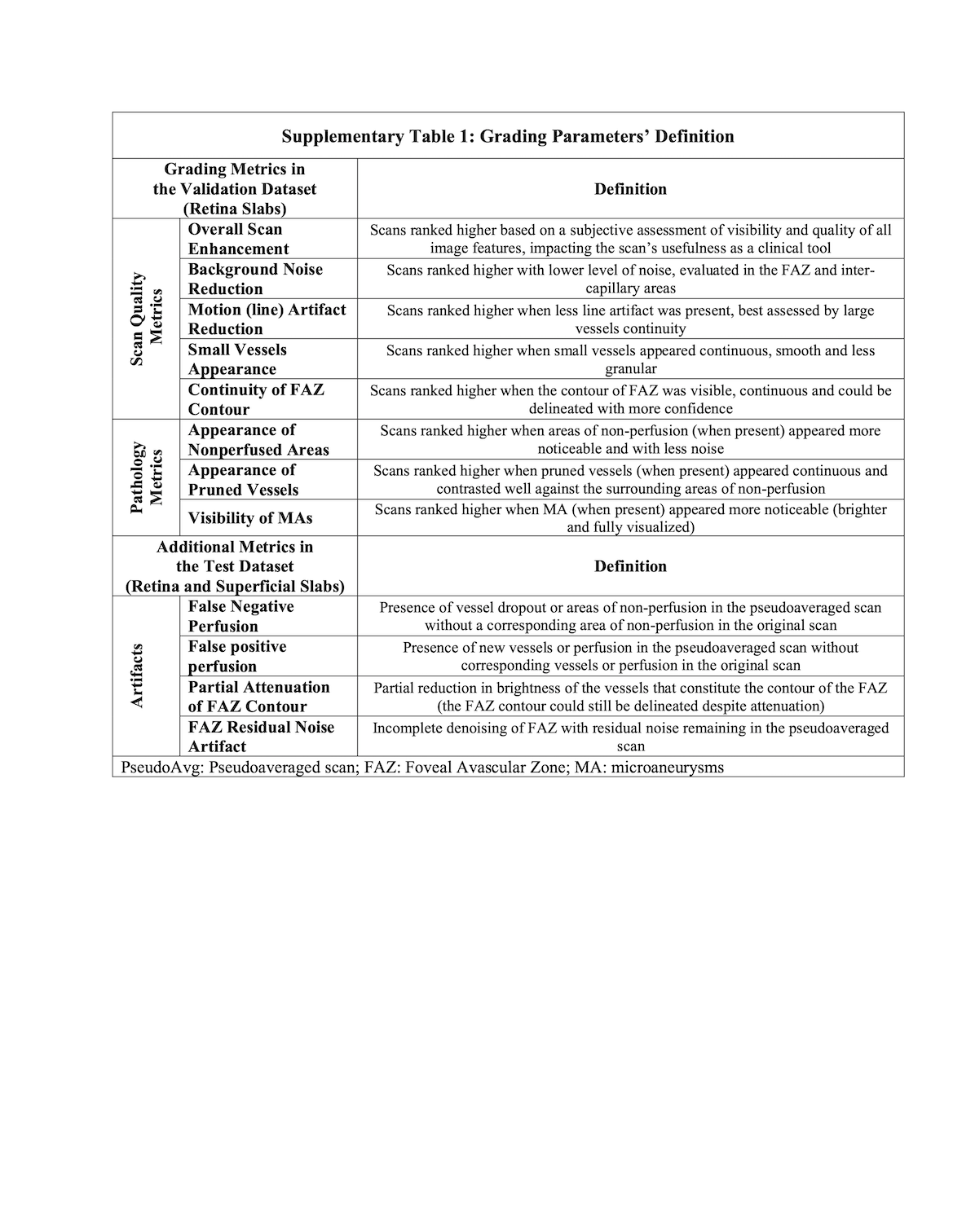

Supplement: Supplementary file 2 — Additional file 1: Table S1. Grading parameter's definition. [file 40942_2023_486_MOESM2_ESM.tiff]
